# Pimozide Inhibits Type II but Not Type I Hair Cells in Chicken Embryo and Adult Mouse Vestibular Organs

**DOI:** 10.3390/biomedicines12122879

**Published:** 2024-12-18

**Authors:** Roberta Giunta, Giulia Cheli, Giorgio Rispoli, Giancarlo Russo, Sergio Masetto

**Affiliations:** 1 Department of Brain and Behavioral Sciences, University of Pavia, 27100 Pavia, Italy; roberta.giunta@unipv.it (R.G.); giulia.cheli01@universitadipavia.it (G.C.); giancarlo.russo@unipv.it (G.R.); 2 Department of Neuroscience and Rehabilitation, University of Ferrara, 44121 Ferrara, Italy; giorgio.rispoli@unife.it

**Keywords:** pimozide, antipsychotic drug, hair cell, vestibular, patch clamp, ionic currents, potassium channels, balance, dizziness

## Abstract

Background: Pimozide is a conventional antipsychotic drug of the diphenylbutylpiperidine class, widely used for treating schizophrenia and delusional disorders and for managing motor and phonic tics in Tourette’s syndrome. Pimozide is known to block dopaminergic D2 receptors and various types of voltage-gated ion channels. Among its side effects, dizziness and imbalance are the most frequently observed, which may imply an effect of the drug on the vestibular sensory receptors, the hair cells. Amniotes possess two classes of vestibular hair cells, named type I and type II hair cells, which differ in terms of signal processing and transmission. We previously reported that Pimozide [3 μM] significantly increased a delayed outward rectifying K^+^ current (*I*_K,V_). Methods and Results: In the present study, using the whole-cell patch-clamp technique we additionally show that Pimozide decreases the inward rectifying K^+^ current (*I*_K,1_) and the mixed Na^+^/K^+^ current (*I*_h_) of chicken embryo type II hair cells, whereas it does not affect type I hair cells’ ionic currents. Since ion channels’ expression can vary depending on age and animal species, in the present study, we also tested Pimozide in adult mouse vestibular hair cells. We found that, like in the chicken embryo, Pimozide significantly increases *I*_K,V_ and decreases *I*_K,1_ and *I*_h_ in type II hair cells. However, in the adult mouse, Pimozide also slightly increased the outward rectifying K^+^ current in type I hair cells. Conclusions: While providing a possible explanation for the vestibular side effects of Pimozide in humans, its inhibitory action on mammalian hair cells might be of interest for the local treatment of vestibular disorders characterized by altered vestibular input, like Ménière’s disease.

## 1. Introduction

Pimozide is an antipsychotic drug primarily used in the therapy for schizophrenia, delusional disorders, and Tourette’s syndrome [1,2]. Despite its widespread use, Pimozide has several severe adverse effects, including extrapyramidal symptoms (such as tremors and rigidity), tardive dyskinesia (involuntary movements), and QT prolongation (a heart rhythm disorder). The mechanisms behind Pimozide’s therapeutic and adverse effects remain only partially understood. Pimozide has been postulated to act at the CNS primarily by blocking dopamine D2 receptors [3]. However, Pimozide also blocks voltage-gated K^+^ and Ca^2+^ channels in various cell types [4,5,6,7,8,9]. Since vestibular problems (dizziness and loss of balance control) are among the most common side effects of Pimozide [10], we have investigated the effect of Pimozide on vestibular hair cells. Amniotes have two types of vestibular sensory cells, named type I and type II hair cells, which differ in morphology, electrophysiological properties, and innervation [11]. We recently reported that Pimozide [3 μM] in chicken embryo vestibular type II hair cells significantly increases a slow delayed rectifier K^+^ conductance (*G*_K,V_), while it weakly blocks a rapid transient outward rectifier K^+^ conductance (*G*_K,A_) [12]. In the first part of the present study, we extended our analysis of Pimozide’s effect on chicken embryo type II hair cells to the inward rectifier K^+^ conductance (*G*_K,1_) and to the mixed Na^+^/K^+^ inward rectifier (*G*_h_). Moreover, we investigated the effect of Pimozide on type I hair cells. Type I hair cells distinctively express *G*_K,L_, a large outward rectifier K^+^ conductance characterized by a hyperpolarized activation threshold (−100 mV; [13,14,15]). We found that Pimozide inhibits *G*_h_ and *G*_K,1_ in type II hair cells, whereas it has no effect on *G*_K,L_ in type I hair cells. Also, Pimozide does not affect the voltage-dependent Na^+^ conductance (*G*_Na_) expressed by some, presumably immature, type I hair cells.

The chicken embryo is an advantageous model for investigating vestibular hair cells because it is easily accessible, and because different ion channels are acquired progressively during prenatal development, which eases the discrimination of their electrophysiological and pharmacological properties. Moreover, the pattern of ion channels expressed by type I and type II hair cells at the time of hatching resembles that of adult birds [16,17]. In rodents, in contrast, the pattern of ion channels changes during neonatal development and cannot be considered mature until the third postnatal week ([18,19,20,21,22]; reviewed in [23]). Moreover, although the general properties of the principal ionic conductances look similar in avians and rodents, differences in their pharmacological properties cannot be excluded *a priori*. Therefore, in the second part of the present study, we investigated the effect of Pimozide on adult mouse vestibular type I and type II hair cells.

We found that Pimozide had a similar effect on mature mouse type II hair cells as it did in chicken embryos; that is, it inhibited *G*_K,1_ and *G*_h_ while it increased *G*_K,V_. The final effect was a significant increase in the outward K^+^ current at −50 mV and above, consistent with hair cell hyperpolarization in the operating voltage range of the receptor. In contrast, in adult mouse type I hair cells, Pimozide exerted a slight agonistic action on the outward K^+^ current. The latter is likely due to Pimozide’s effect on the small *I*_K,V_ which is present, together with *I*_K,L_, in mouse type I hair cells [24].

The inhibitory action of Pimozide on mammalian vestibular hair cells, besides providing an explanation for Pimozide’s side effects, may be of therapeutic interest for those vestibular diseases involving altered vestibular sensory input.

## 2. Materials and Methods

### 2.1. Surgical Dissection

Experiments were performed on hair cells in whole vestibular epithelia dissected from chicken embryos between embryonic day (E)14 and E21 and C57BL/6J mice from postnatal day 22 (P22) to P365. This study was conducted according to the guidelines of the Declaration of Helsinki. Animal procedures on chicken embryos conform with the guidelines from Directive 2010/63/EU of the European Parliament on the protection of animals used for scientific purposes. The study protocol did not require the authorization of the Ministry of Health as indicated in the Legislative Decree 4 March 2014, n. 26, following the implementation of Directive (2010)/63/EU on the protection of animals used for scientific purposes. As far as experiments on mice are concerned, the study protocol (PT252) was approved by the Italian Ministry of Health on 13 May 2024.

Details of the dissection procedure in the chicken embryo have been reported in Giunta et al. [12]. Briefly, once removed from the eggs, embryos were decapitated following brief anesthesia with 2-Bromo-2-Chloro-1,1,1-trifluoroethane (Halothane), and semicircular canals or utricles were dissected in chilled extracellular solution (in mM): 145 NaCl, 3 KCl, 2 CaCl_2_, 0.6 MgCl_2_, 5.6 D-glucose, 15 HEPES; pH 7.4 with NaOH; osmolality ~310 mOsm∙kg^−1^). Data from utricle and crista hair cells were pooled since no differences were observed.

For mice, after cervical dislocation, the head was sectioned in two halves along the sagittal plane, the brain removed, and the bony labyrinth located. To preserve cell viability, the two half heads were transferred to a Petri dish filled with chilled extracellular solution (in mM): 135 NaCl, 5.8 KCl, 1.3 CaCl_2_, 0.9 MgCl_2_, 0.7 NaH_2_PO_4_, 5.6 D-glucose, 10 HEPES-NaOH. Sodium pyruvate (2 mM), amino acids, and vitamins were added from concentrates (Thermo Fisher Scientific, Loughborough, UK). The pH was adjusted to 7.4 (osmolality ~308 mmol∙kg^−1^).

To isolate the utricle and the three *ampullae*, surgical dissection was continued under a stereomicroscope. The dissected organ to be investigated was then immobilized at the bottom of the recording chamber by a nylon mesh glued to a silver ring. Hair cells were viewed using an upright microscope (Zeiss 2 FS plus, Göttingen, Germany) equipped with a 67X water immersion objective. Hair cells in the sensory organs were approached from their basolateral membrane. To ease access to the chosen hair cell, cellular debris was cleared by using the tip of the patch pipette, while a second pipette was then used to record.

### 2.2. Whole-Cell Patch-Clamp Recordings

Whole-cell patch-clamp experiments were conducted at room temperature (22 °C) using an Axopatch 200B amplifier (Molecular Devices, San Josè, CA, USA). Voltage protocol application and data acquisition were controlled by pClamp 10.3 software using a Digidata 1440A board (Molecular Devices, San Josè, CA, USA). Voltage-clamp recordings were low-pass filtered at 5 kHz (4-pole Bessel) and sampled at 50 kHz. Data analysis was performed using Clampfit (Molecular Devices, San Josè, CA, USA) and OriginPro 9.0 software (OriginLab, Northampton, MA, USA).

Patch pipettes (4–7 MΩ) were pulled either from Kovar capillaries (Hilgenberg, Malsfeld, Germany) or borosilicate glass capillaries (World Precision Instruments, Hitchin, UK).

Potassium currents in chicken embryo hair cells were recorded using a KCl-based intracellular solution containing the following (in mM): 134 KCl, 2 MgCl_2_, 1 CaCl_2_, 11 EGTA, and 10 HEPES. pH was adjusted to 7.4 using KOH (osmolality: 290 mOsm∙kg^−1^). For mouse experiments, the intracellular solution was as follows (in mM): 131 KCl, 3 MgCl_2_, 1 EGTA, 5 HEPES, 5 Na_2_ATP, and 10 Na_2_Phosphocreatine. pH was adjusted to 7.4 using KOH (osmolality: 290 mOsm∙kg^−1^).

The voltages presented in the figures and text were not adjusted for the liquid junction potential with the intracellular solution (resulting in a 3 mV negative potential inside the pipette); hence, nominal voltages are reported. No subtraction of leakage current was performed during the experiments nor offline.

### 2.3. Pimozide

Pimozide (Tocris, Bristol, UK) was initially dissolved in DMSO and then introduced into the extracellular solution to achieve a final concentration of 3 μM/L. This concentration was chosen to maximize comparison of its effects with those reported in the relevant literature in mammalian (including human heterologously expressed) voltage-gated K^+^ channels [5,25,26]. In our previous study in chicken embryo type II hair cells, we also tested a concentration of 0.3 µM and showed an overall milder effect of Pimozide 0.3 µM on *G*_K,V_ compared to 3 µM [12].

Pimozide was perfused by a multi-barreled pipette positioned close (a few mm) to the preparation. To quantify Pimozide’s effect, each cell included in this study was first recorded in control conditions and then exposed to Pimozide. Current responses before and after Pimozide perfusion was analyzed offline. The preparation was changed after each perfusion to avoid recording from cells that had already been exposed to Pimozide.

### 2.4. Data Analysis and Statistical Methods

The equilibrium potential for K^+^ (*E*_K_) was calculated according to the Nernst equation:

(1) $$E_{K}=RT/F\left(\ln\left({\left[K^{+}\right]}_{out}/{\left[K^{+}\right]}_{in}\right)\right)$$

where the subscripts ‘‘out” and ‘‘in” refer to the extracellular and intracellular solution, respectively, and were −96 mV for the chicken embryo experiments and −81 mV for the mouse experiments.

Statistical analysis was performed by Prism GraphPad 6.0 Software (San Diego, CA, USA). Following D’Agostino and Pearson and Shapiro–Wilk normality tests, statistical comparison of current means was performed by paired Student’s *t*-test (two-tailed) and Wilcoxon matched-pairs signed rank test for parametric and non-parametric data, respectively. Statistical comparison of time-to-peak and decay time constant was performed by Mann–Whitney test.

All numerical values, degrees of freedom, and statistical values (df, t, U and W), in addition to the *p*-values, are listed in the Supplementary Material. In the text, n = number of cells and mean values are quoted as means ± standard error (S.E.). In all figures, the level of statistically significant difference is as follows: * *p* ≤ 0.05; ** *p* ≤ 0.01; *** *p* ≤ 0.001; **** *p* ≤ 0.0001.

## 3. Results

### 3.1. Preliminary Remarks

**Figure 1:**
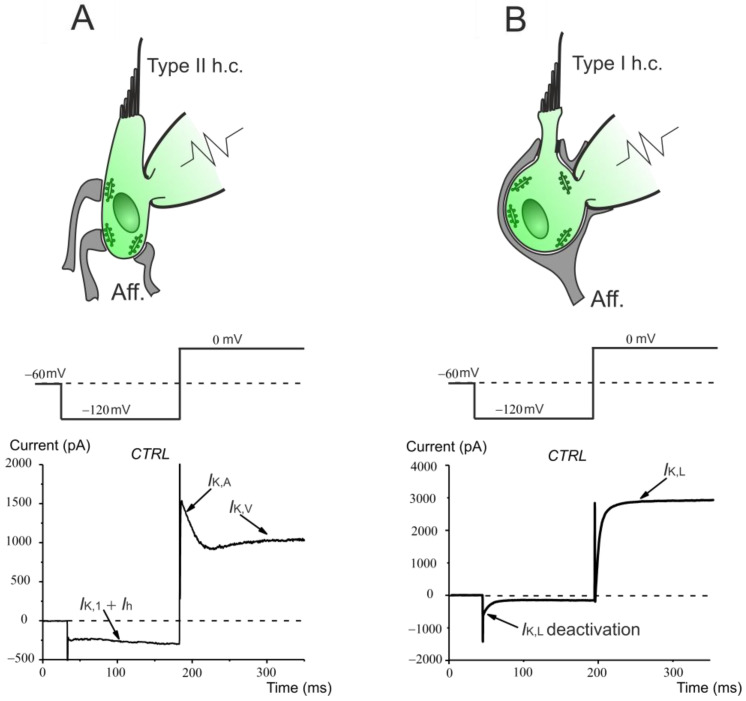
Schematic drawing of the typical morphological and electrophysiological features of mature type I and type II hair cells. The typical voltage protocol used to identify the hair cell type in whole-cell patch-clamp recordings is shown above the current responses (bottom panels). (**A**) Each type II hair cell is contacted by several bouton-type afferent nerve terminals, each facing a presynaptic ribbon that tethers numerous glutamate-containing vesicles. Whole-cell recordings reveal the expression of inward rectifying K^+^ (*I*_K,1_) and mixed Na^+^/K^+^ (*I*_h_) current in response to hyperpolarizing voltage steps and of the transient (*I*_K,A_) and sustained (*I*_K,V_) outward rectifying K^+^ currents, which activate around −60 mV. (**B**) Each type I hair cell is contacted by a large afferent nerve terminal, called a calyx, facing several presynaptic ribbons, which encloses the basolateral membrane of hair cell. Whole-cell recordings show the expression of a low-voltage activated outward rectifying K^+^ current; *I*_K,L_, which is fully activated at −60 mV, deactivates completely at voltage more hyperpolarized than −100 mV and activates at 0 mV (note the absence of inactivation at this depolarized voltage).

Besides their different morphology and innervation, in whole-cell patch-clamp experiments, vestibular type I and type II hair cells can be identified by their pattern of ion channel expression (Figure 1). Usually, starting from a membrane holding voltage (*V*_hold_) close to the hair cells’ resting membrane potential (about −60 mV), hair cells are first hyperpolarized to reveal the presence of the inward rectifying currents *I*_K,1_ and/or *I*_h_ and then depolarized to elicit *I*_K,V_ and, when present, *I*_K,A_ (Figure 1A). By contrast, in type I hair cells, hyperpolarization produces a deactivation of *I*_K,L_, which is fully active at −60 mV, while depolarization allows to appreciate the *I*_K,L_ activation time course (Figure 1B).

In the following results, we will show how Pimozide affects the different ionic conductances in chicken embryo and adult mouse vestibular type I and type II hair cells.

### 3.2. Pimozide Inhibits Inward Rectifier Conductances in Chicken Embryo Type II Hair Cells

Current responses were recorded from 27 chicken embryo type II hair cells, which were then classified into three groups according to the inward rectifier conductances expressed.

**Figure 2:**
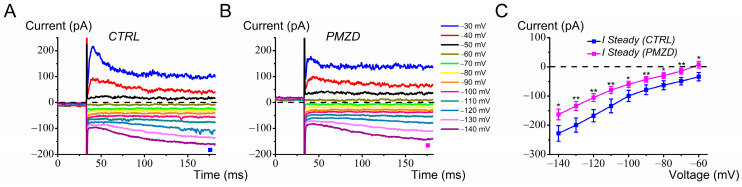
Effect of Pimozide [3 μM] on chicken embryo type II hair cells expressing *I*_h_. Cells were held at *V*_hold_ of −60 mV and then iteratively voltage-stepped for 150 ms at voltages between −140 mV and −30 mV in 10 mV increments. Here and in the next figures, CTRL = control condition; PMZD = Pimozide; horizontal dashed line = zero-current level. (**A**), control current; (**B**), Pimozide. The filled squares indicate the time points at which the steady-state current was measured. Legend in panel B also applies to panel A. (**C**), average inward rectifying steady-state current (*I*_h_) measured between −140 mV and −60 mV (n = 14). Values are shown as mean ± S.E.; see Table S1 in the Supplementary Material. * *p* ≤ 0.05; ** *p* ≤ 0.01.

Several type II hair cells investigated showed the mixed Na^+^/K^+^ conductance *G*_h_ in response to hyperpolarization from *V*_hold_ of −60 mV. Pimozide [3 μM] reduced *I*_h_ at all membrane voltages from −60 mV to −140 mV (Figure 2A,B). Three depolarizing voltage steps (−50 mV, −40 mV, and −30 mV) have also been included in Figure 2A,B to show that, as mentioned in the Introduction, Pimozide also reduced the fast transient outward rectifying K^+^ current, *I*_K,A_, while it increased the slow sustained outward rectifying K^+^ current, *I*_K,V_, in the voltage range near the cell resting membrane potential (~−60 mV; [16]). The average current–voltage (*I*-*V*) relationship for the steady-state inward rectifying current, *I*_h_, is shown in Figure 2C. The decrease in *I*_h_ produced by Pimozide was statistically significant at all voltages. Note that, following Pimozide, the inward current at −60 mV turned outward, which is due to the combined decrease in *I*_h_, a cationic inward current which reverses near −40 mV [16,27], and the increase in *I*_K,V_.

**Figure 3:**
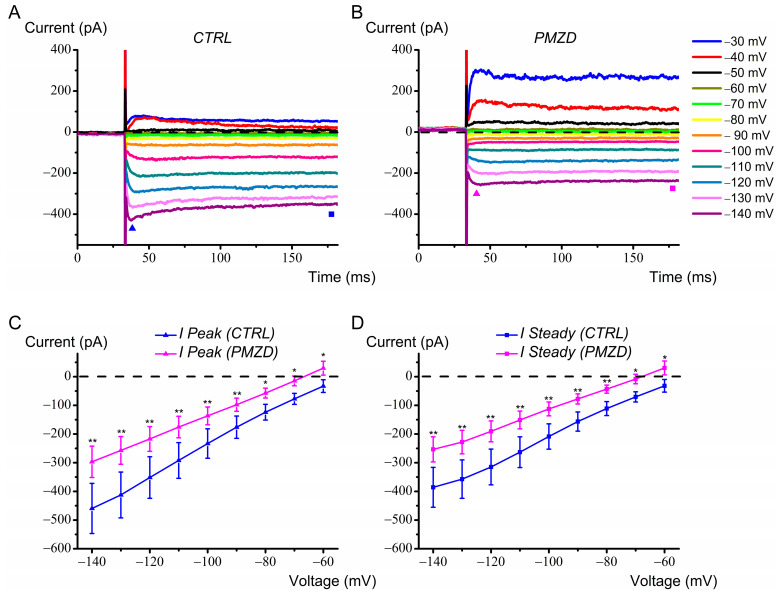
Effect of Pimozide [3 μM] on chicken embryo type II hair cells expressing *I*_K,1_. Cells were held at *V*_hold_ of −60 mV and then iteratively voltage-stepped for 150 ms at voltages between −140 mV and −30 mV in 10 mV increments. (**A**), control current and (**B**), Pimozide. The filled triangles indicate the time points at which the peak current was measured. The steady-state current was measured towards the end of the depolarizing steps, as indicated by the filled squares. Legend in panel B also applies to panel A. (**C**), average peak and (**D**), steady-state current (*I*_K,1_) measured between −140 mV and −60 mV (n = 8). Values are shown as mean ± S.E.; see Tables S2 and S3 in the Supplementary Material. * p ≤ 0.05; ** p ≤ 0.01.

Some type II hair cells predominantly expressed a large fast inward rectifier K^+^ conductance, *G*_K,1_. Pimozide significantly reduced *I*_K,1_ at all voltages (Figure 3). Note that, since *I*_K,1_ reverses at the K^+^ equilibrium potential [28], which in the current experimental conditions is estimated to be −96 mV (see Section 2), *I*_K,1_ is expected to be outward at *V*_hold_ of −60 mV. Instead, the macroscopic current was inward at *V*_hold_ in most cells showing *I*_K,1_. This likely results from the combination of several factors: (1) the outward component of *I*_K,1_ should be very small since *I*_K,1_ in vestibular hair cells is carried by the strong rectifier Kir2.1 conductance [29,30]; (2) traces were not subtracted for the leakage current (see Section 2); (3) a small contribution from *I*_h_ might have been present. Note that the inward current at *V*_hold_ turned outward following Pimozide perfusion, which is likely due to its strong agonistic effect on *I*_K,V_ (compare the depolarized voltage steps in Figure 3A,B).

In some type II hair cells, both *I*_h_ and *I*_K,1_ were clearly present and reduced by Pimozide (see Figure S1 in the Supplementary Material).

**Figure 4:**
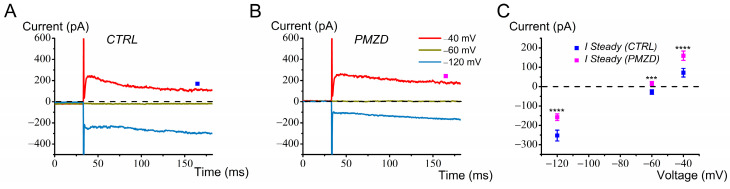
Pimozide [3 μM] reduces the inward rectifying current at hyperpolarized voltages while it increases the outward rectifying K^+^ current in chicken embryo type II hair cells. Cells were held at V_hold_ of −60 mV and stepped to −60 mV or −40 mV. (**A**), control current; (**B**), Pimozide perfusion. The filled squares indicate the time points at which the steady-state current was measured. Legend in panel B also applies to panel A. (**C**), average steady-state current (n = 27) measured at −120 mV, −60 mV and −40 mV. Values are shown as mean ± S.E.; see Table S5 in the Supplementary Material. *** *p* ≤ 0.001; **** *p* ≤ 0.0001.

The above results can be summarized as in Figure 4, which shows the average effect of Pimozide on all chicken embryo type II hair cells investigated (n = 27), at three representative voltages as indicated in figure. The substantial increase in the outward K^+^ current at −60 mV and −40 mV is consistent with Pimozide hyperpolarizing type II hair cells at rest and in the range of the receptor potential, as also shown in previously reported current-clamp experiments [12].

### 3.3. Pimozide Does Not Affect Chicken Embryo Type I Hair Cells

**Figure 5:**
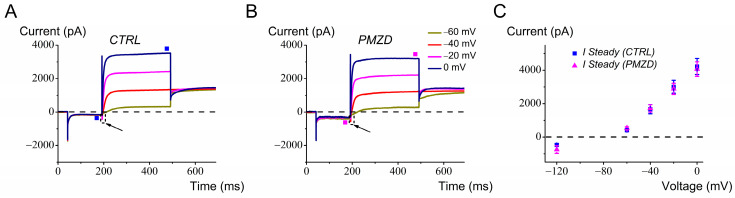
Pimozide [3 μM] does not affect chicken embryo type I hair cells. Cells were held at *V*_hold_ of −70 mV and conditioned at −120 mV for 150 ms before iteratively stepping from −60 mV to 0 mV. (**A**), control current. (**B**), Pimozide perfusion. In both panels, the arrows point at the Na^+^ current (see text). The filled squares indicate the time points at which the steady-state current was measured. Legend in panel B also applies to panel A. (**C**), average steady-state *I*/*V* relation (n = 9). Values are shown as mean ± S.E.; see Table S6 in the Supplementary Material. No significant differences were found at any voltage.

The signature conductance of type I hair cells, *G*_K,L_, appears late during development (around E17; [16]). *G*_K,L_ is by far the predominant conductance in type I hair cells. It is fully activated at −60 mV and does not inactivate, while it is completely deactivated at voltages more negative than 100 mV. We recorded the response to Pimozide from nine type I hair cells. As shown in the representative traces in Figure 5A,B, no significant effect on the macroscopic currents was found following Pimozide perfusion. A small transient Na^+^ current (*I*_Na_) was also detectable (arrow), which is dealt with in the next section. The average effect of Pimozide is shown at a few representative voltages (−120 mV, −60 mV, −40 mV, −20 mV and 0 mV; Figure 5C). Since chicken embryo type I hair cells exhibit negligible inward rectifying currents [16], only the hyperpolarizing potential of −120 mV is shown—note that only the deactivating (tail) *I*_K,L_ is visible upon hyperpolarization from *V*_hold_ (−70 mV) to −120 mV. The lack of effect of Pimozide in type I hair cells indicates that *G*_K,L_ is unaffected by this drug.

### 3.4. Pimozide Does Not Affect I_Na_ in Chicken Embryo Type I Hair Cells

**Figure 6:**
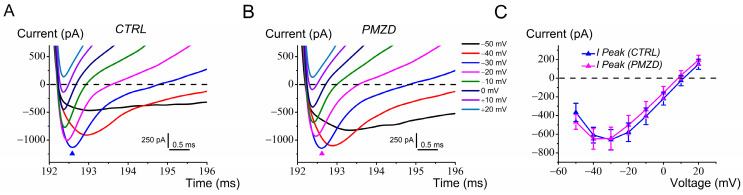
Pimozide [3 μM] does not affect *I*_Na_ in chicken embryo type I hair cells. Cells were held at *V*_hold_ of −70 mV and then conditioned at −120 mV before stepping to the voltages shown in figure. (**A**), control current. (**B**), Pimozide perfusion. The filled triangles indicate the time points at which the peak (*I*_Na_) current was measured. Legend in panel B also applies to panel A. (**C**), average peak current (n = 6) measured at the different test potentials. Values are shown as mean ± S.E.; see Table S7 in the Supplementary Material. No significant differences were found at any voltage.

Consistent with a previous report [17], most (6 out of 9) type I hair cells exhibited a fast transient *I*_Na_. *I*_Na_ is also present in most immature type II hair cells [17], but we do not report it here because the voltage protocol for type II hair cells did not include the hyperpolarized conditioning step at −120 mV needed to remove *I*_Na_ inactivation [17]. Conditioning at −120 mV on the other hand was required for type I hair cells to fully deactivate *I*_K,L_ before its activation with depolarization. Note that *I*_Na_ could be seen, despite its relatively small amplitude compared to *I*_K,L_, because of its much faster kinetics at less depolarized voltages. However, as shown in Figure 6A,B, which show a zoom of the region delimited by the dashed rectangle in Figure 5A,B, *I*_Na_ could not be discerned +10 mV and above due to contamination by the outward potassium currents. As shown by the average peak current/voltage relation in Figure 6C, Pimozide did not affect the amplitude of *I*_Na_. For example, the average amplitude of *I*_Na_ at −30 mV was −659 pA (±108 pA) in control condition vs. −644 pA (±119 pA) after Pimozide administration (n = 6; E19–20; t = 0.1900, df = 5, *p* = 0.8568 Student’s paired *t* test).

The above experiments in the chicken embryo show that Pimozide substantially increases the outward K^+^ current in type II hair cells, whereas it does not affect type I hair cells.

In the next section, we present data concerning the effect of Pimozide on adult mouse vestibular type II and type I hair cells.

### 3.5. Pimozide’s Effects on Adult Mouse Type II Hair Cells

**Figure 7:**
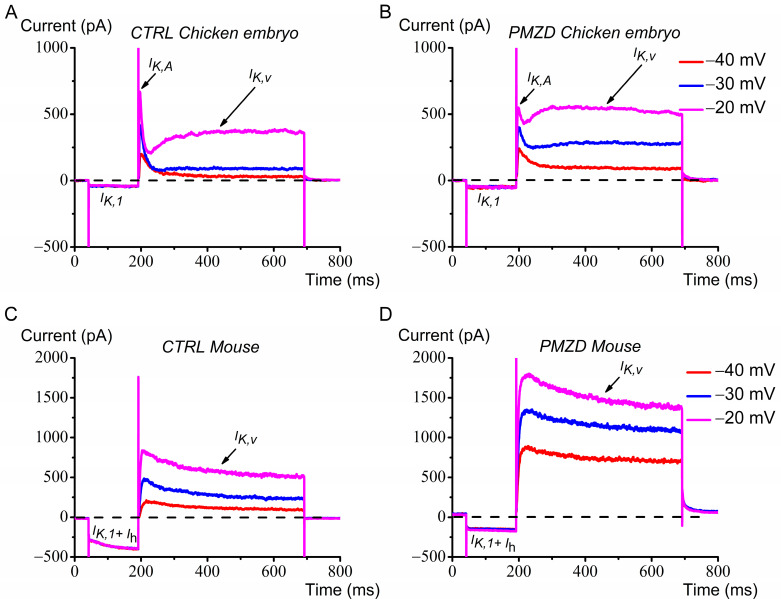
Comparison of Pimozide’s effect on chicken embryo and adult mouse type II hair cells. (**A**,**B**): macroscopic currents recorded from a representative chicken embryo type II hair cell in response to the voltage steps of −40 mV, −30 mV, and −20 mV after conditioning at −120 mV from *V*_hold_ of −70 mV before and after Pimozide perfusion, respectively. (**C**,**D**): current response to the same experimental protocol in a representative adult mouse type II hair cell. Legend in panels B,D also applies to panels A,C.

Previous [12] and present experiments in chicken embryo type II hair cells show that Pimozide slightly decreases the fast transient outward rectifying K^+^ current *I*_K,A_, significantly increases the delayed outward rectifying K^+^ current *I*_K,V_, which results from shifting its activation curve towards more hyperpolarized voltages [12], and decreases the inward rectifying currents *I*_K,1_ and *I*_h_. These results are summarized in Figure 7A,B for easy comparison with the effect of Pimozide on adult mouse type II hair cells (Figure 7C,D).

**Figure 8:**
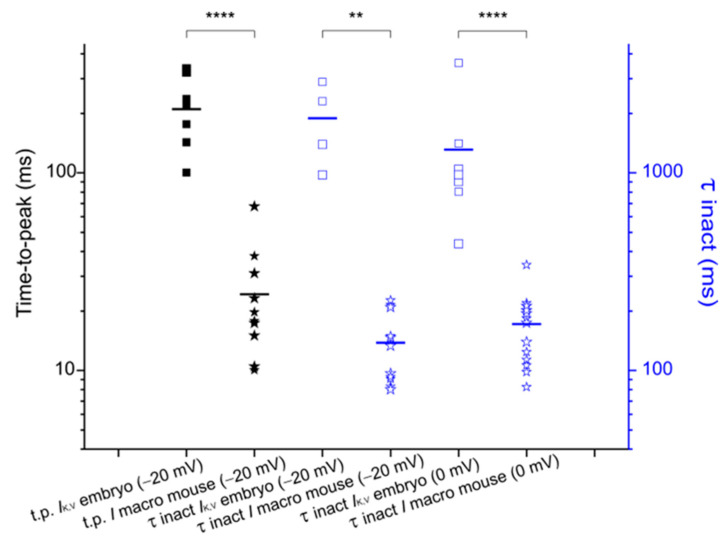
Comparison of outward K^+^ current kinetics between chicken embryo and adult mouse type II hair cells. Black vertical axis and symbols: time-to-peak (t.p.) of the macroscopic outward K^+^ current recorded from the chicken embryo (filled squares) and adult mouse (filled stars) type II hair cells at −20 mV. Blue vertical axis and symbols: inactivation time constant (τ) of *I*_K,V_ (empty squares) and decay τ of the macroscopic outward K^+^ current (empty stars) at −20 mV and at 0 mV. The horizontal bar indicates the mean value for each distribution of data points, which are plotted on a logarithmic scale. Values are shown as mean ± S.E.; see Tables S8 and S9 in the Supplementary Material. ** *p* ≤ 0.01; **** *p* ≤ 0.0001.

Like in the chicken embryo, following Pimozide administration the inward rectifying currents *I*_h_ and *I*_K,1_ decreased, while the sustained outward rectifying K^+^ current increased. Unlike the chicken embryo, however, the peak outward K^+^ current did not decrease following Pimozide, which seems related to the absence of the fast *I*_K,A_ (e.g., compare Figure 7A,C). The lack of the fast *I*_K,A_ in adult mouse type II hair cells is consistent with previous reports showing its expression in frog and avian vestibular type II hair cells, but not in rodents (for a review see [31]). The question arises whether, given the similar agonistic effect of Pimozide on the sustained outward K^+^ current, the macroscopic K^+^ current in adult mouse type II hair cells is analogous to the *I*_K,V_ in chicken embryo type II hair cells. The monotonic decay of the macroscopic outward current recorded from mouse type II hair cells may in fact indicate the presence of a single population of K^+^ channels identifiable as the K,V channels. To this purpose, we compared (Figure 8) the time-to-peak and the decay time constant of the macroscopic outward K^+^ current recorded at −20 mV from mouse type II hair cells, with the time-to peak and the inactivation time constant of *I*_K,V_ recorded from chicken embryo type II hair cells at −20 mV following conditioning at −40 mV, which inactivated most *I*_K,A_ [12]. We found that the time-to-peak of the isolated *I*_K,V_ expressed by chicken embryo type II hair cells (0.21 s ± 0.03 s; n = 8) was significantly different from that of the macroscopic K^+^ current recorded from mouse type II hair cells (0.024 s ± 0.005 s; n = 11) (U = 0, *p* < 0.0001 Mann–Whitney test). Similarly, the inactivation time constant of the isolated *I*_K,V_ in chicken embryo type II hair cells (1.9 s ± 0.4 s; n = 4) was significantly different from the decay time constant of the macroscopic K^+^ current recorded from the mouse type II hair cells (0.14 s ± 0.02 s; n = 11) (U = 0, *p* = 0.0015 Mann–Whitney test). Note that at −20 mV the isolated *I*_K,V_ showed inactivation, and therefore could be fitted, in only half of the cells. We therefore compared the inactivation kinetics even at 0 mV, at which voltage *I*_K,V_ inactivated in all type II hair cells from the chicken embryo, and we found that the difference was highly significant. The inactivation time constant of the isolated *I*_K,V_ at 0 mV was 1.3 s (±0.4 s; n = 7) vs. a decay time constant of the macroscopic K^+^ current in mouse type II hair cells of 0.19 s (±0.02 s; n = 11) (U = 0, *p* < 0.0001 Mann–Whitney test). Thus, despite a similar agonistic effect of Pimozide on *I*_K,V_ expressed by the chicken embryo and adult mouse type II hair cells, the different kinetics indicate some differences concerning K,V channel molecular composition and/or modulation by accessory subunits.

**Figure 9:**
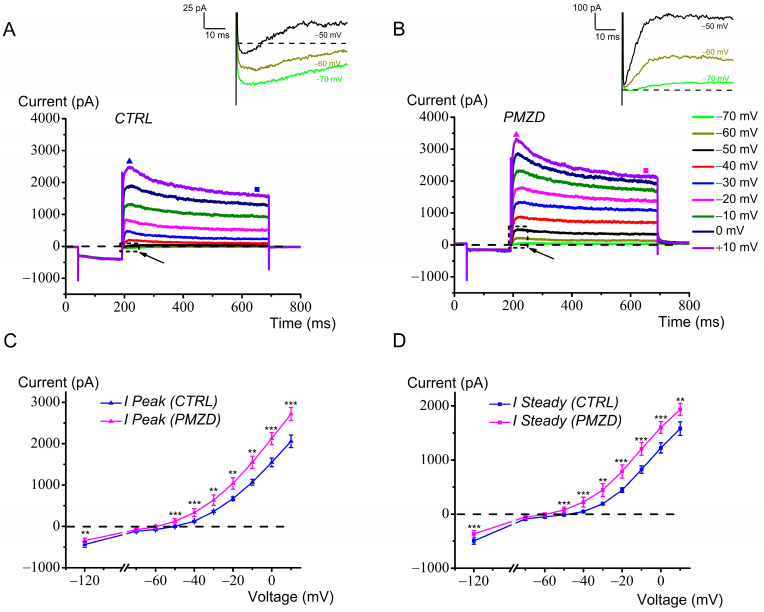
Pimozide [3 μM] increases the outward K^+^ current in adult mouse type II hair cells. (**A**,**B**): representative currents recorded in response to voltage steps between −70 mV and 10 mV, in 10 mV increments, following conditioning at −120 mV for 150 ms from a *V*_hold_ of −70 mV. In both panels (**A**,**B**), the arrows point to the small inward currents elicited upon return to −70 mV, −60 mV, and −50 mV after conditioning at −120 mV. The corresponding expanded traces framed by the dashed rectangles (time window = 65 ms) are shown enlarged in the upper panels (note the different vertical scales). The filled triangles indicate the time points at which the peak current was measured. The steady-state current was measured towards the end of the depolarizing steps, as indicated by the filled squares. Legend in panel B also applies to panel A. (**C**,**D**): average peak and steady-state *I*–*V* relations obtained from adult mouse type II hair cells with and without Pimozide (n = 11). Values are shown as mean ± S.E.; see Tables S10 and S11 in the Supplementary Material. ** *p* ≤ 0.01; *** *p* ≤ 0.001.

The whole family of outward K^+^ currents recorded from the adult mouse type II hair cells is shown in Figure 9A,B. Note that the tail of *I*_h_ is responsible for the initial transient inward current at −70 mV, −60 mV and −50 mV, which is turned to outward following Pimozide (see also the zoomed inset figures). Thus, the increase in the outward current near the resting membrane potential of the hair cell is produced by a combination of *I*_K,V_ increase and *I*_h_ inhibition.

Differently from the chicken embryo type II hair cells, in which, following Pimozide, *I*_K,V_ increased only up to −20 mV and then decreased for more depolarized voltages [12], here, the peak and steady-state outward K^+^ currents increased even at the most depolarized voltages. The double effect of Pimozide on chicken embryo *I*_K,V_ (increase or decrease at less or more depolarized voltages) could be explained by Pimozide shifting both its activation curve and kinetics toward more hyperpolarized voltages, such that at more depolarized voltages the strong acceleration of *I*_K,V_ inactivation kinetics resulted in a decrease in its amplitude [12]. This effect of Pimozide on the inactivation kinetics is not as much pronounced in mouse type II hair cells, which is presumably why the outward K^+^ current is increased at all voltages. The average peak and steady-state *I*-*V* relations for the macroscopic outward K^+^ current, before and after Pimozide perfusion, are shown in Figure 9C,D. The increase in the peak and sustained outward K^+^ current was significant at all depolarized voltages.

### 3.6. Pimozide Only Slightly Affects Adult Mouse Type I Hair Cells

**Figure 10:**
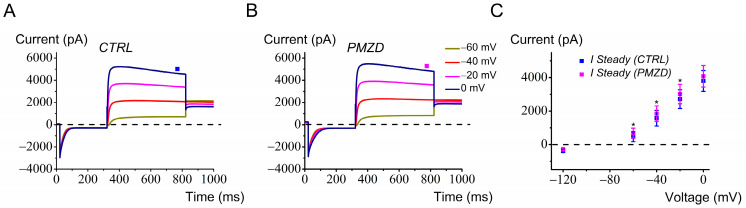
Effect of Pimozide [3 μM] on adult mouse type I hair cells. Cells were held at −70 mV, conditioned at −120 mV for 300 ms before iteratively stepping from −60 mV to 0 mV (20 mV increment), and finally stepped to −40 mV before returning to −70 mV. (**A**), control current. (**B**), Pimozide perfusion. Note that at the most depolarized potentials of −20 mV and 0 mV, a decay in the amplitude of the outward current is observed, indicating some K^+^ accumulation in the calyceal synaptic clef [24,37]. The steady-state current was measured towards the end of the depolarizing steps, as indicated by the filled squares. Legend in panel B also applies to panel A. (**C**), average steady-state *I*–*V* relationships obtained from adult mouse type I hair cells before and after Pimozide perfusion (n = 5). Values are shown as mean ± S.E.; see Table S12 in the Supplementary Material. * *p* ≤ 0.05.

A major difficulty in whole-cell recording from adult mouse type I hair cells was represented by *G*_K,L_ variability, an aspect not so critical in chicken embryos. *I*_K,L_ showed either run-down or run-up in different cells. As previously noted in neonatal and juvenile rat type I hair cells [21], the voltage dependence of *G*_K,L_ varies with time during the recording, presumably because of the change in some diffusible factors following dialysis of the intracellular solution by the patch pipette solution. *G*_K,L_ has been reported to be modulated by intracellular cGMP [32,33], consistent with *G*_K,L_ involving the Kv1.8 subunit [34], which has a cyclic nucleotide binding domain [35]. An additional cause of *G*_K,L_ variability could be the residual afferent nerve calyx, which remains attached to the hair cell basolateral membrane even after piercing by the patch pipette tip [24]. It has been shown that the inner membrane of the calyx (facing the synaptic cleft) is connected to the hair cells basolateral membrane by a septate-like junction [36]. It is presumable that, during the recording, a progressive degeneration of the damaged calyx occurs, which might mechanically deform the attached presynaptic membrane and affect *G*_K,L_. We never observed such a phenomenon with type II hair cells. Also, we did not observe such a variability in nor distortion of *G*_K,L_ with time in chicken embryo type I hair cells, perhaps because the immature afferent calyx is not yet tightly attached to the hair cell. Therefore, to reduce the duration of the recording, we tested the effect of Pimozide at only a few selected voltage steps, and we only considered those recordings where the amplitude of *I*_K,L_ was sufficiently constant before Pimozide perfusion. The results obtained in this way are shown in Figure 10.

We found a significant, though modest, increase in the outward K^+^ current at −60 mV, −40 mV, and −20 mV (*p* < 0.05) following Pimozide treatment.

## 4. Discussion

Present results show that Pimozide affects several types of ion channels in vestibular hair cells. In chicken embryo type II hair cells, Pimozide showed an antagonistic effect on the inward rectifier conductances *G*_h_ and *G*_K,1_. Because of the strong agonistic effect on *G*_K,V, however,_ the net effect was an increase in the outward K^+^ current in the range of the receptor potential. Results obtained from adult mouse type II hair cells point to a similar conclusion. Since an increase in the outward K^+^ current will produce a hyperpolarization of the hair cell membrane, which will close the voltage-gated Ca^2+^ channels coupled to glutamate exocytosis, Pimozide is expected to inhibit signaling from adult mammalian type II hair cells.

Concerning type I hair cells, Pimozide appeared ineffective in chicken embryos, while it produced a modest increase in the outward K^+^ current in adult mouse type I hair cells. The final effect of Pimozide on signal transmission from type I hair cells is, however, difficult to anticipate, since both conventional (quantal, glutamatergic) and non-quantal transmission occurs at the hair cell–calyx synapse [38,39,40]. The small increase in K^+^ outflow produced by Pimozide should hyperpolarize type I hair cells and therefore slightly decrease glutamate release like in type II hair cells. On the other hand, the (limited) increase in K^+^ content in the synaptic cleft might directly depolarize the calyx afferent.

An interesting aspect not considered in our study concerns the regional expression of ion channels (see, e.g., [31] for a recent review), which might be associated with the predominance of *I*_h_ or *I*_K,1_ and/or *I*_K,A_ which we found in different hair cells. Unfortunately, we were not able to correlate different sets of conductances with hair cell position in the organ, possibly because hair cells were recorded at different depths in the sensory organs. It is likely that a longitudinal or transverse section of the sensory epithelia would be better suited to this aim, as previously reported (see, e.g., [41]).

As far as the molecular nature of the ion channels targeted by Pimozide is concerned, *Kcnj2* (Kir2.1) and *Hcn1* are expressed in all chicken (at postnatal day 7, P7) and mouse type II hair cells [42,43]. Therefore, they are the likely target of Pimozide inhibitory action on the inward rectifying currents *I*_K1_ and *I*_h_, respectively.

More difficult to identify are the subunits responsible for the outward rectifying K^+^ currents. In mice, constitutive knockout of *Kcna10*, which encodes for the channel-pore-forming subunit K_V_1.8, abolished *I*_K,L_ in type I hair cells, *I*_K,A_ in type II hair cells, and most *I*_K,V_ in type II-hair cells of the utricle [34]. On the basis of the above results, and since recent single-cell expression studies on mouse utricle type I hair cells have detected just one K_V_1 subunit, the K_V_1.8 [44,45,46], *G*_K,L_ has been hypothesized to consist of a K_V_1.8 tetra-homomer [34]. Even chicken type I hair cells express the *Kcna10* transcript [42,46]. Given the very similar kinetics and voltage dependence of *I*_K,L_ in the chicken embryo and adult mouse (e.g., compare Figure 5 and Figure 10 here), it is likely that even in chicken embryo type I hair cells *G*_KL_ is produced by a homotetramer of the K_V_1.8 subunit. The lack of effect of Pimozide in chicken embryo type I hair cells, and its modest effect on the macroscopic K^+^ current expressed by adult mouse type I hair cells, which is largely dominated by *I*_K,L_, suggests that the K_V_1.8 subunit is insensitive to Pimozide. The modest increase in the macroscopic outward K^+^ current in adult mouse type I hair cells might in fact be due to Pimozide’s agonistic effect on *G*_K,V_, which is also expressed in adult mouse type I hair cells [14,15,18,24,37].

As far as the molecular nature of *G*_K,V_ is concerned, the residual sustained outward K^+^ current in K_V_1.8^−/−^ mouse type I and type II hair cells was blocked by XE991, indicating that it involves the K_V_7 channel subunit [34]. The K_V_7.2 subunit is expressed in the chicken utricle type I and type II hair cells at P7 [42], although whether it is also present in chicken embryo hair cells remains to be ascertained. Also, *G*_K,V_ showed a slower activation/inactivation kinetics in chicken embryo compared to adult mouse type II hair cells, suggesting a possible difference in subunit composition and/or modulation by accessory proteins. It seems interesting to note that the action of Pimozide on K,V channels reported here resembles that of retigabine on K_V_7.2-7.5 channels, since it similarly increases the outward K^+^ current by shifting the activation curve towards more hyperpolarized voltages [47].

Concerning the fast *I*_K,A_ found in chicken embryo type II hair cells, which was reduced by Pimozide [12], we found no evidence for it in adult mouse type II hair cells, consistent with no inhibition of the peak outward K^+^ current by Pimozide. The K_V_1.4 subunit has been shown to drive the fast *I*_K,A_ in adult pigeon type II hair cells [48]. The K_V_1.4 subunit is the only K_V_1 subunit (Kv1.1 to Kv1.8) which, when expressed as a homomer, has complete N-type (fast) inactivation [49]. Since the K_V_1.4 subunit is abundantly expressed in mammalian vestibular type II hair cells [44,46], it is possible that heteromers of K_V_1.4 with other K_V_1 subunits produce a slowly inactivating K^+^ current which could contribute to *I*_K,V_. Further studies of Pimozide’s effects on transgenic mice missing specific ion channel subunits would greatly help in the identification of its targets in vestibular hair cells.

In conclusion, the present study demonstrates that Pimozide produces a significant increase in the sustained outward K^+^ current expressed by type II hair cells. The presumable consequence in vivo would be a decrease in the sensory vestibular input from type II hair cells to the CNS. This effect might be at least in part responsible for the common vestibular side effects of Pimozide.

On the other hand, a drug able to turn down the vestibular input might be of interest for those peripheral vestibular disorders where there is excessive/altered vestibular input, like in Ménière’s disease. Local application to the inner ear could be considered (see [50]) to prevent other collateral effects, especially considering that Pimozide has not been reported to affect hearing. It is also of interest that novel Pimozide derivatives that show little binding affinity to dopamine D2 receptors, thus theoretically avoiding related side effects like motor dysfunctions, have been recently developed [51]. It would be interesting to test whether these derivatives maintain the agonistic effect of Pimozide on hair cells’ K^+^ channels.

## Data Availability

Data can be made available upon direct request to the corresponding author.

## Supplementary Materials

The following supporting information can be downloaded at https://www.mdpi.com/article/10.3390/biomedicines12122879/s1, Figure S1: Effect of Pimozide [3 µM] on chicken embryo type II hair cells expressing both *I*_h_ and *I*_K,1_; Table S1: *I*_h_ steady-state values from chicken embryo type II hair cells; Table S2: *I*_K,1_ peak values from chicken embryo type II hair cells; Table S3: *I*_K,1_ steady-state values from chicken embryo type II hair cells; Table S4: Steady-state values of the inward rectifying current of chicken embryo type II hair cells; Table S5: Steady-state values of the inward rectifying current of chicken embryo type II hair cells; Table S6: *I*_K,L_ steady-state values from chicken embryo type I hair cells; Table S7: *I*_Na_ peak values from chicken embryo type I hair cells; Table S8: Comparison of outward K^+^ current time-to-peak between chicken embryo and adult mouse type II hair cells; Table S9: Comparison of outward K^+^ current decay time constant between chicken embryo and adult mouse type II hair cells; Table S10: Peak values of the macroscopic K^+^ current from adult mouse type II hair cells; Table S11: Steady-state values of the macroscopic K^+^ current from adult mouse type II hair cells; Table S12: *I*_K,L_ steady-state values from adult mouse type I hair cells.
