# Supplementary material for: Pimozide Inhibits Type II but Not Type I Hair Cells in Chicken Embryo and Adult Mouse Vestibular Organs
Source: Biomedicines. 2024 Dec 18;12(12):2879. doi: 10.3390/biomedicines12122879 (PMC11673355; doi:10.3390/biomedicines12122879)
Supplement: Supplementary file 1 [file biomedicines-12-02879-s001.zip › biomedicines-3367342-supplementary.pdf]

SUPPLEMENTARY MATERIAL

Figure S1: Pimozide [3 μM] effect on chicken embryo type-II hair cells expressing both I_h and $I_{K,1}$. Cells were held at V_{hold} of −60 mV and then iteratively voltage-stepped for 150 ms at voltages between −140 mV and −30 mV in 10 mV increments. CTRL = control condition; PMZD = Pimozide; horizontal dashed line = zero-current level. (A), control current; (B), Pimozide. The filled squares indicate the time points at which the steady-state current was measured. (C), average inward rectifying steady-state current (Ih) measured between −140 mV and −60 mV (n = 14). Values are shown as mean ±S.E.; see Table S4. * $p \leq 0.05$.

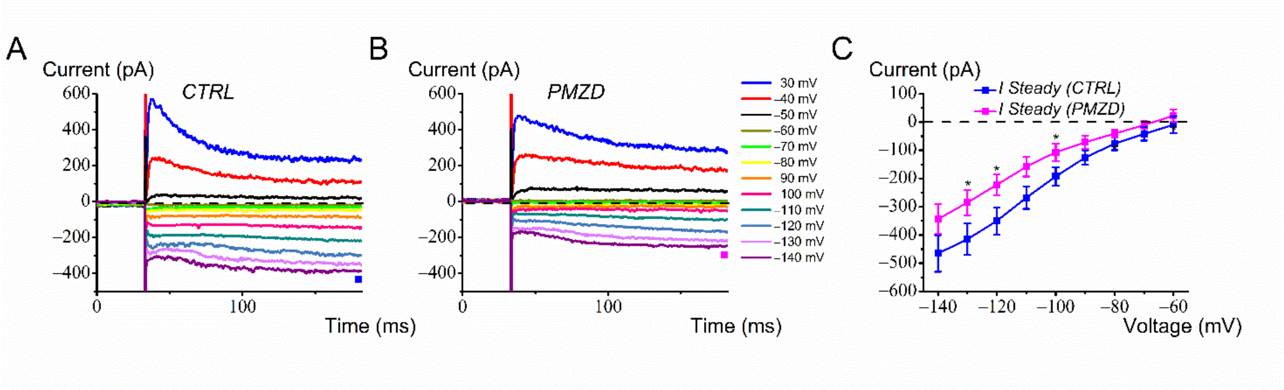

Table S1: I_h steady-state values from chicken embryo type-II hair cells. Average steady-state current obtained at the voltage steps listed from 14 chicken embryo type-II hair cells (E15-21) expressing the mixed Na^+/K^+ current (I_h), before (CTRL) and after (PMZD) perfusion of pimozide [3 μM]. Values are shown as mean ± S.E.; the level of statistically significant difference is as follow: * $p \leq 0.05$; ** $p \leq 0.01$; *** $p \leq 0.001$; **** $p \leq 0.0001$.

I steady				
Vm	I (CTRL)	I (PMZD)		
−60 mV	−0.03 nA ± 0.01 nA	0.01 nA ± 0.01 nA	*	t = 2.671, df = 13, p-value = 0.0192 Student's paired t test
−70 mV	−0.05 nA ± 0.01 nA	−0.01 nA ± 0.01 nA	**	W = 81.00, p-value = 0.0085 Wilcoxon signed-rank test
−80 mV	−0.06 nA ± 0.02 nA	−0.03 nA ± 0.01 nA	*	W = 75.00, p-value = 0.0166 Wilcoxon signed-rank test
−90 mV	−0.08 nA ± 0.02 nA	−0.04 nA ± 0.01 nA	**	W = 83.00, p-value = 0.0067 Wilcoxon signed-rank test
−100 mV	−0.10 nA ± 0.02 nA	−0.06 nA ± 0.01 nA	*	W = 73.00, p-value = 0.0203 Wilcoxon signed-rank test
−110 mV	−0.13 nA ± 0.02 nA	−0.08 nA ± 0.01 nA	**	W = 83.00, p-value = 0.0067 Wilcoxon signed-rank test
−120 mV	−0.17 nA ± 0.02 nA	−0.11 nA ± 0.01 nA	**	W = 89.00, p-value = 0.0031 Wilcoxon signed-rank test
−130 mV	−0.20 nA ± 0.02 nA	−0.13 nA ± 0.02 nA	**	W = 89.00, p-value = 0.0031 Wilcoxon signed-rank test
−140 mV	−0.23 nA ± 0.03 nA	−0.16 nA ± 0.02 nA	*	W = 77.00, p-value = 0.0134 Wilcoxon signed-rank test

Table S2: $I_{K,1}$ peak values from chicken embryo type-II hair cells. Average peak current obtained at the voltage steps listed from 8 chicken embryo type-II hair cells (E14-21) expressing the inward (anomalous) rectifying current $I_{K,1}$, before (CTRL) and after (PMZD) perfusion of pimozide [3 µM]. Values are shown as mean ± S.E.; the level of statistically significant difference is as follow: * $p \leq 0.05$; ** $p \leq 0.01$; *** $p \leq 0.001$; **** $p \leq 0.0001$.

**I peak**				
V_m	I (CTRL)	I (PMZD)		
−60 mV	−0.03 nA ± 0.02 nA	0.03 nA ± 0.02 nA	*	t = 2.983, df = 7, p-value = 0.0204 Student's paired t test
−70 mV	−0.08 nA ± 0.02 nA	−0.01 nA ± 0.02 nA	*	t = 3.374, df = 7, p-value = 0.0119 Student's paired t test
−80 mV	−0.12 nA ± 0.03 nA	−0.06 nA ± 0.02 nA	*	t = 3.497, df = 7, p-value = 0.0100 Student's paired t test
−90 mV	−0.18 nA ± 0.04 nA	−0.10 nA ± 0.02 nA	**	t = 3.624, df = 7, p-value = 0.0085 Student's paired t test
−100 mV	−0.23 nA ± 0.05 nA	−0.14 nA ± 0.03 nA	**	W = 36.00, p-value = 0.0078 Wilcoxon signed-rank test
−110 mV	−0.29 nA ± 0.06 nA	−0.18 nA ± 0.04 nA	**	W = 36.00, p-value = 0.0078 Wilcoxon signed-rank test
−120 mV	−0.35 nA ± 0.07 nA	−0.22 nA ± 0.04 nA	**	t = 3.800, df = 7, p-value = 0.0067 Student's paired t test
−130 mV	−0.41 nA ± 0.08 nA	−0.26 nA ± 0.05 nA	**	t = 4.141, df = 7, p-value = 0.0043 Student's paired t test
−140 mV	−0.46 nA ± 0.09 nA	−0.30 nA ± 0.05 nA	**	t = 4.152, df = 7, p-value = 0.0043 Student's paired t test

Table S3: $I_{K,1}$ steady-state values from chicken embryo type-II hair cells. Average steady-state current obtained at the voltage steps listed from 8 chicken embryo type-II hair cells (E14-21) expressing the inward (anomalous) rectifying current $I_{K,1}$, before (CTRL) and after (PMZD) perfusion of pimozide [3 µM]. Values are shown as mean ± S.E.; the level of statistically significant difference is as follow: * $p \leq 0.05$; ** $p \leq 0.01$; *** $p \leq 0.001$; **** $p \leq 0.0001$.

**I steady**				
V_m	I (CTRL)	I (PMZD)		
−60 mV	−0.03 nA ± 0.02 nA	0.03 nA ± 0.02 nA	*	t = 2.950, df = 7, p-value = 0.0214 Student's paired t test
−70 mV	−0.07 nA ± 0.02 nA	−0.01 nA ± 0.02 nA	*	t = 3.312, df = 7, p-value = 0.0129 Student's paired t test
−80 mV	−0.11 nA ± 0.02 nA	−0.04 nA ± 0.01 nA	**	W = 36.00, p-value = 0.0078 Wilcoxon signed-rank test
−90 mV	−0.16 nA ± 0.03 nA	−0.08 nA ± 0.02 nA	**	t = 3.537, df = 7, p-value = 0.0095 Student's paired t test
−100 mV	−0.21 nA ± 0.04 nA	−0.11 nA ± 0.02 nA	**	W = 36.00, p-value = 0.0078 Wilcoxon signed-rank test
−110 mV	−263.24 nA ± 0.05 nA	−0.15 nA ± 0.03 nA	**	W = 36.00, p-value = 0.0078 Wilcoxon signed-rank test
−120 mV	−0.31 nA ± 0.06 nA	−0.19 nA ± 0.04 nA	**	t = 3.881, df = 7, p-value = 0.0060 Student's paired t test
−130 mV	−0.36 nA ± 0.07 nA	−0.23 nA ± 0.04 nA	**	t = 4.152, df = 7, p-value = 0.0043 Student's paired t test
−140 mV	−0.39 nA ± 0.07 nA	−0.25 nA ± 0.04 nA	**	t = 3.966, df = 7, p-value = 0.0054 Student's paired t test

Table S4: Steady-state values of the inward rectifying current of chicken embryo type-II hair cells. Average steady-state current obtained at the voltage steps listed from 6 chicken embryo type-II hair cells (E16-21) expressing both the inward (anomalous) rectifying current $I_{K,1}$ and the mixed Na^+/K^+ current I_h, before (CTRL) and after (PMZD) perfusion of pimozide [3 μM]. Values are shown as mean ± S.E.; the level of statistically significant difference is as follow: * $p \le 0.05$; ** $p \le 0.01$; *** $p \le 0.001$; **** $p \le 0.0001$.

**I steady**				
V_m	I (CTRL)	I (PMZD)		
−60 mV	−0.01 nA ± 0.03 nA	0.02 nA ± 0.02 nA	ns	t = 0.7879, df = 5, p-value = 0.4664<br>Student's paired t test
−70 mV	−0.04 nA ± 0.02 nA	−0.01 nA ± 0.01 nA	ns	W = 9.000, p-value = 0.4375<br>Wilcoxon signed-rank test
−80 mV	−0.08 nA ± 0.02 nA	−0.04 nA ± 0.01 nA	ns	W = 15.00, p-value = 0.1563<br>Wilcoxon signed-rank test
−90 mV	−0.13 nA ± 0.03 nA	−0.07 nA ± 0.02 nA	ns	t = 2.304, df = 5, p-value = 0.0694<br>Student's paired t test
−100 mV	−0.20 nA ± 0.03 nA	−0.11 nA ± 0.03 nA	*	t = 2.617, df = 5, p-value = 0.0473<br>Student's paired t test
−110 mV	−0.27 nA ± 0.04 nA	−0.16 nA ± 0.03 nA	ns	W = 19.00, p-value = 0.0625<br>Wilcoxon signed-rank test
−120 mV	−0.35 nA ± 0.05 nA	−0.22 nA ± 0.04 nA	*	t = 2.793, df = 5, p-value = 0.0383<br>Student's paired t test
−130 mV	−0.41 nA ± 0.06 nA	−0.29 nA ± 0.04 nA	*	t = 2.813, df = 5, p-value = 0.0374<br>Student's paired t test
−140 mV	−0.47 nA ± 0.06 nA	−0.34 nA ± 0.05 nA	ns	t = 2.132, df = 5, p-value = 0.0862<br>Student's paired t test

Table S5: Steady-state values of the inward rectifying current of chicken embryo type-II hair cells. Average steady-state current obtained at three hyperpolarizing voltages from 27 chicken embryo type-II hair cells (E14-21) expressing both I_h and $I_{K,1}$, before (CTRL) and after (PMZD) perfusion of pimozide [3 μM]. Values are shown as mean ± S.E.; the level of statistically significant difference is as follow: * $p \le 0.05$; ** $p \le 0.01$; *** $p \le 0.001$; **** $p \le 0.0001$.

**I steady**				
V_m	Iof the inwar (CTRL)	I (PMZD)		
−40 mV	0.07 nA ± 0.02 nA	0.16 nA ± 0.02 nA	****	W = 324.0, p-value <0.0001<br>Wilcoxon signed-rank test
−60 mV	−0.03 nA ± 0.01 nA	0.02 nA ± 0.01 nA	***	W = 276.0, p-value = 0.0005<br>Wilcoxon signed-rank test
−120 mV	−0.25 nA ± 0.03 nA	−0.16 nA ± 0.02 nA	****	W = 350.0, p-value <0.0001<br>Wilcoxon signed-rank test

Table S6: $I_{K,L}$ steady-state values from chicken embryo type-I hair cells. Average steady-state current obtained at the voltage steps listed from 9 type-I hair cells (E19-21), before (CTRL) and after (PMZD) perfusion of pimozide [3 µM]. Values are shown as mean ± S.E.; the level of statistically significant difference is as follow: * $p \leq 0.05$; ** $p \leq 0.01$; *** $p \leq 0.001$; **** $p \leq 0.0001$.

**I steady**				
V_m	I (CTRL)	I (PMZD)		
0 mV	4.2 nA ± 0.5 nA	4.1 nA ± 0.4 nA	ns	t = 0.7121, df = 8, p-value = 0.4966<br>Student's paired t test
−20 mV	3.0 nA ± 0.4 nA	2.9 nA ± 0.3 nA	ns	W = −11.00, p-value = 0.5703<br>Wilcoxon signed-rank test
−40 mV	1.7 nA ± 0.3 nA	1.7 nA ± 0.2 nA	ns	W = 5.000, p-value = 0.8203<br>Wilcoxon signed-rank test
−60 mV	0.41 nA ± 0.09 nA	0.52 nA ± 0.09 nA	ns	t = 1.309, df = 8, p-value = 0.2270<br>Student's paired t test
−120 mV	−0.47 nA ± 0.08 nA	−0.7 nA ± 0.2 nA	ns	t = 1.487, df = 8, p-value = 0.1753<br>Student's paired t test

Table S7: I_{Na} peak values from chicken embryo type-I hair cells. Average peak values of the Na^+ current recorded at the voltage steps listed from 6 type-II hair cells (E15-21), before (CTRL) and after (PMZD) perfusion of pimozide [3 µM]. Values are shown as mean ± S.E.; the level of statistically significant difference is as follow: * $p \leq 0.05$; ** $p \leq 0.01$; *** $p \leq 0.001$; **** $p \leq 0.0001$.

**I peak**				
V_m	I (CTRL)	I (PMZD)		
40 mV	0.47 nA ± 0.08 nA	0.53 nA ± 0.09 nA	ns	W = 7.000, p-value = 0.5625<br>Wilcoxon signed-rank test
30 mV	0.31 nA ± 0.06 nA	0.34 nA ± 0.07 nA	ns	W = 3.000, p-value = 0.8438<br>Wilcoxon signed-rank test
20 mV	0.15 nA ± 0.05 nA	0.19 nA ± 0.05 nA	ns	t = 0.7750, df = 5, p-value = 0.4734<br>Student's paired t test
10 mV	−0.03 nA ± 0.05 nA	0.03 nA ± 0.05 nA	ns	t = 0.9955, df = 5, p-value = 0.3652<br>Student's paired t test
0 mV	−0.22 nA ± 0.07 nA	−0.16 nA ± 0.07 nA	ns	t = 1.067, df = 5, p-value = 0.3346<br>Student's paired t test
−10 mV	−0.4 nA ± 0.1 nA	−0.34 nA ± 0.09 nA	ns	t = 1.217, df = 5, p-value = 0.2780<br>Student's paired t test
−20 mV	−0.6 nA ± 0.1 nA	−0.5 nA ± 0.1 nA	ns	t = 1.375, df = 5, p-value = 0.2275<br>Student's paired t test
−30 mV	−0.7 nA ± 0.1 nA	−0.6 nA ± 0.1 nA	ns	t = 0.1900, df = 5, p-value = 0.8568<br>Student's paired t test
−40 mV	−0.61 nA ± 0.08 nA	−0.7 nA ± 0.1 nA	ns	t = 0.4438, df = 5, p-value = 0.6757<br>Student's paired t test
−50 mV	−0.4 nA ± 0.1 nA	−0.47 nA ± 0.08 nA	ns	t = 0.8985, df = 5, p-value = 0.4101<br>Student's paired t test

Table S8: Comparison of outward K+ current time-to-peak between chicken embryo and adult mouse type-II hair cells. Average time-to-peak of the macroscopic K+ current recorded at -20 mV from 8 chicken embryo (E15-19) and 11 adult mouse (P22-365) type-II hair cells. Values are shown as mean ± S.E.; the level of statistically significant difference is as follow: * $p \le 0.05$; ** $p \le 0.01$; *** $p \le 0.001$; **** $p \le 0.0001$.

Time to Peak				
V_m	$I_{K,v}$ embryo	*I* macro mouse		
−20 mV	0.21 s ± 0.03 s	0.024 s ± 0.005 s	****	U = 0, p-value <0.0001 Mann Whitney test

Table S9: Comparison of outward K+ current decay time constant between chicken embryo and adult mouse type-II hair cells. Average inactivation time constant (τ) of the outward K+ current recorded at -20 mV from 4 chicken embryo (E15-18) and 11 adult mouse (P22-365) type-II hair cells, and at 0 mV from 7 chicken embryo (E15-19) and 14 adult mouse (P22-365) type-II hair cells. Values are shown as mean ± S.E.; the level of statistically significant difference is as follow: * $p \le 0.05$; ** $p \le 0.01$; *** $p \le 0.001$; **** $p \le 0.0001$.

Decay time constant				
V_m	$I_{K,v}$ embryo	*I* macro mouse		
0 mV	1.9 s ± 0.4 s	0.14 s ± 0.02 s	****	U = 0, p-value <0.0001 Mann Whitney test
−20 mV	1.3 s ± 0.4 s	0.17 s ± 0.02 s	**	U = 0, p-value = 0.0015 Mann Whitney test

Table S10: Peak values of the macroscopic K+ current from adult mouse type-II hair cells. Average peak current obtained at the voltage steps listed from 11 type-II hair cells of the adult mouse (P21-365), before (CTRL) and after (PMZD) perfusion of pimozide [3 μM]. Values are shown as mean ± S.E.; the level of statistically significant difference is as follow: * $p \le 0.05$; ** $p \le 0.01$; *** $p \le 0.001$; **** $p \le 0.0001$.

I peak				
V_m	I (CTRL)	I (PMZD)		
10 mV	2.1 nA ± 0.2 nA	2.7 nA ± 0.2 nA	***	t = 4.635, df = 10, p-value = 0.0009 Student's paired t test
0 mV	1.5 nA ± 0.1 nA	2.1 nA ± 0.1 nA	***	t = 5.791, df = 10, p-value = 0.0002 Student's paired t test
−10 mV	1.07 nA ± 0.07 nA	1.5 nA ± 0.1 nA	**	t = 4.396, df = 10, p-value = 0.0013 Student's paired t test
−20 mV	0.67 nA ± 0.04 nA	1.0 nA ± 0.1 nA	**	W = 62.00, p-value = 0.0029 Wilcoxon signed-rank test
−30 mV	0.36 nA ± 0.03 nA	0.6 nA ± 0.1 nA	**	W = 64.00, p-value = 0.0020 Wilcoxon signed-rank test
−40 mV	0.12 nA ± 0.02 nA	0.3 nA ± 0.1 nA	***	W = 66.00, p-value = 0.0010 Wilcoxon signed-rank test
−50 mV	0.00 nA ± 0.02 nA	0.12 nA ± 0.06 nA	***	W = 66.00, p-value = 0.0010 Wilcoxon signed-rank test
−60 mV	−0.08 nA ± 0.02 nA	−0.01 nA ± 0.04 nA	ns	t = 2.189, df = 10, p-value = 0.0534 Student's paired t test
−70 mV	−0.12 nA ± 0.03 nA	−0.08 nA ± 0.03 nA	ns	t = 2.170, df = 10, p-value = 0.0551 Student's paired t test
−120 mV	−0.44 nA ± 0.07 nA	−0.34 nA ± 0.06 nA	**	t = 3.392, df = 10, p-value = 0.0069 Student's paired t test

Table S11: Steady-state values of the macroscopic K^+ current from adult mouse type-II hair cells. Average steady-state current obtained at the voltage steps listed from 11 type-II hair cells of the adult mouse (P21-365), before (CTRL) and after (PMZD) perfusion of pimozide [3 µM]. Values are shown as mean ± S.E.; the level of statistically significant difference is as follow: * $p \leq 0.05$; ** $p \leq 0.01$; *** $p \leq 0.001$; **** $p \leq 0.0001$.

**I steady**				
V_m	I (CTRL)	I (PMZD)		
10 mV	1.6 nA ± 0.1 nA	1.9 nA ± 0.1 nA	**	t = 4.427, df = 10, p-value = 0.0013 Student's paired t test
0 mV	1.2 nA ± 0.1 nA	1.6 nA ± 0.1 nA	***	t = 6.083, df = 10, p-value = 0.0001 Student's paired t test
−10 mV	0.82 nA ± 0.07 nA	1.2 nA ± 0.1 nA	***	t = 4.932, df = 10, p-value = 0.0006 Student's paired t test
−20 mV	0.44 nA ± 0.04 nA	0.8 nA ± 0.1 nA	***	W = 66.00, p-value = 0.0010 Wilcoxon signed-rank test
−30 mV	0.19 nA ± 0.02 nA	0.4 nA ± 0.1 nA	**	W = 64.00, p-value = 0.0020 Wilcoxon signed-rank test
−40 mV	0.05 nA ± 0.01 nA	0.22 nA ± 0.09 nA	***	W = 66.00, p-value = 0.0010 Wilcoxon signed-rank test
−50 mV	−0.01 nA ± 0.02 nA	0.08 nA ± 0.05 nA	***	W = 66.00, p-value = 0.0010 Wilcoxon signed-rank test
−60 mV	−0.05 nA ± 0.03 nA	−0.01 nA ± 0.03 nA	ns	W = 44.00, p-value = 0.0537 Wilcoxon signed-rank test
−70 mV	−0.09 nA ± 0.03 nA	−0.05 nA ± 0.03 nA	ns	W = 42.00, p-value = 0.0674 Wilcoxon signed-rank test
−120 mV	−0.50 nA ± 0.06 nA	−0.37 nA ± 0.06 nA	***	t = 4.986, df = 10, p-value = 0.0005 Student's paired t test

Table S12: $I_{K,L}$ steady-state values from adult mouse type-I hair cells. Average steady-state current obtained at the voltage steps listed from 5 type-I hair cells (P24-87), before (CTRL) and after (PMZD) perfusion of pimozide [3 µM]. Values are shown as mean ± S.E.; the level of statistically significant difference is as follow: * $p \leq 0.05$; ** $p \leq 0.01$; *** $p \leq 0.001$; **** $p \leq 0.0001$.

**I steady**				
V_m	I (CTRL)	I (PMZD)		
0 mV	3.8 nA ± 0.6 nA	4.1 nA ± 0.6 nA	ns	t = 2.748, df = 4, p-value = 0.0515 Student's paired t test
−20 mV	2.7 nA ± 0.6 nA	3.0 nA ± 0.6 nA	*	t = 3.036, df = 4, p-value = 0.0385 Student's paired t test
−40 mV	1.6 nA ± 0.5 nA	1.8 nA ± 0.5 nA	*	t = 3.145, df = 4, p-value = 0.0347 Student's paired t test
−60 mV	0.5 nA ± 0.3 nA	0.7 nA ± 0.3 nA	*	t = 4.412, df = 4, p-value = 0.0116 Student's paired t test
−120 mV	−0.4 nA ± 0.1 nA	−0.29 nA ± 0.07 nA	ns	t = 1.844, df = 4, p-value = 0.1390 Student's paired t test
